# Psychometric properties and network analysis of the Chinese version of the uncertainty about disease and treatment scale for patients undergoing hemodialysis

**DOI:** 10.3389/fpsyg.2025.1688355

**Published:** 2026-01-05

**Authors:** Yiru Wang, Tianci Tong, Xinyue Gao, Jing Wu, Jing Chu

**Affiliations:** School of Nursing, Naval Medical University, Shanghai, China

**Keywords:** health psychology, uncertainty, hemodialysis, measurement, network analysis

## Abstract

**Purpose:**

This study aimed to translate and culturally adapt the Uncertainty about Disease and Treatment Scale (UC-D&TS), systematically evaluate its reliability and validity among Chinese hemodialysis patients, and use network analysis to explore the latent structural relationships among constructs, providing tools and theoretical support for precise identification and intervention in the future.

**Methods:**

The study was conducted across five hemodialysis centers in the eastern and northeast part of China. Following Brislin translation model, the original UC-D&TS were developed to Chinese version. EFA was used to clarify the scale structure. Network analysis via EGA examined inter-item associations and centrality features. CFA assessed model fit. Additionally, test-retest reliability, convergent validity, and criterion-related validity were evaluated to comprehensively examine the psychometric properties of the scale.

**Results:**

A total of 404 hemodialysis patients were included. EFA led to the removal of two items with factor loadings below 0.4, resulting in a five-factor structure. EGA confirmed the robustness of the structure and identified core items related to key psychological themes such as crisis preparedness, alternative treatment cognition based on expected influence and strength values. CFA supported a well-fitting second-order model and the scale demonstrated excellent internal consistency. All factors showed average variance extracted values > 0.50 and composite reliability values above 0.70, indicating good psychometric characteristics.

**Conclusion:**

The Chinese version of UC-D&TS exhibits strong reliability and validity in hemodialysis patients. Network analysis reveals meaningful relationships among key psychological constructs, facilitating identification of core areas of patient distress. This scale serves as an effective tool for assessing uncertainty perceptions, offering valuable guidance for clinical psychological support and personalized interventions.

## 1 Introduction

Chronic Kidney Disease (CKD) is clinically defined as persistent abnormalities in kidney structure or function exceeding 3 months, with significant implications for global health outcomes (Kidney Disease: Improving Global Outcomes (Kdigo) Ckd Work Group., 2024). Global epidemiological data indicate that among 218 countries, evidence on CKD prevalence was available in 161 (74%), revealing a median prevalence of 9.5% (Bello et al., 2024). Within China, approximately 82 million adults were diagnosed with CKD in 2023, reflecting an estimated prevalence of 8.2% (Wang et al., 2023). According to the Global Burden of Disease (GBD) report, CKD is projected to become the fifth leading cause of global mortality by 2040 (Foreman et al., 2018). As CKD progresses to end-stage renal disease (ESRD), patients should receive kidney replacement therapy or conservative care to sustain life (Chen et al., 2025). According to the Chinese national renal data system, the number of hemodialysis(HD) patients reached 916,000, with 185,000 new patients added in 2023.

Due to the pressure of long−term therapy and complications, patients receiving HD often suffer from psychological distresses and suffer uncertainty about unforeseen circumstances and future life (Cheng et al., 2022). According to Mishel’s definition, uncertainty in illness is defined as the difficulty in interpreting the significance of events associated with illness or the inability to predict illness outcomes, which contains ambiguity of disease stage, treatment complexity, information deficiency in diagnosis and severity of the disease, as well as the unpredictability of the prognosis (Mishel, 1988). It is a natural existence (Johnson Wright et al., 2009) and can be appraised as an opportunity for positive adaption or as a danger associated with psychological distress (Mishel, 1988). Due to the unpredictable nature of disease progression and complex clinical condition, it is particularly prevalent among patients undergoing HD. Previous studies indicated that Patients undergoing hemodialysis in China have a moderate or high degree of uncertainty in illness in China, which was influenced by education level, job, duration of hemodialysis, social support, etc. (Hong et al., 2019). Based on the results of Kim and Kim (2019) the negative impacts of uncertainty on the perception of and adjustment to HD, including decreasing adherence to treatment regimens (Kim and Kim, 2019). A scoping review was to systematically explore and describe the literature on the link between uncertainty and mental health and found that most studies (79%) reported a positive association between uncertainty and mental health problems (Massazza et al., 2023). A study demonstrated uncertainty in illness is corrected with anxiety, depression positively and with quality of life negatively (Shu and Xiu, 1996). Therefore, it is crucial to focus on the uncertainty of illness in hemodialysis patients.

Accurate measurement of disease and treatment uncertainty is the basis for implementing interventions for hemodialysis patients. Thus far, only one tool has been designed to measure uncertainty about the disease, developed by Mishel (1988), the existing Mishel Illness Uncertainty Scale is categorized into four types based on different measurement subjects: Mishel uncertainty in illness scale for adult (MUIS-A), Mishel uncertainty in illness scale-community form (MUIS-C), Mishel uncertainty in illness scale for family member (MUIS-FM) and Parent’s perception uncertainty in illness scale (PPUS). Among them, the MUIS-A designed by Mishel (1981) contains 30 items covering two dimensions: “multi-attribute ambiguity” and “unpredictability.” It was the only tool at that time specifically measuring illness uncertainty in hospitalized patients and has been applied to assess uncertainty in various chronic diseases (Mishel, 1981). The Taiwanese researcher Xu Shulian et al. translated the MUIS-A (Shu and Xiu, 1996) into Chinese and used it to measure the level of disease uncertainty in Chinese patients in 1997. The Chinese version of the MUIS-A exhibited good reliability and validity. Later, Zeng-Jie et al. (2018) develop the Revised Chinese Version of Mishel Uncertainty in Illness Scale (RC-MUIS), and test its reliability and validity in a sample of Chinese patients with cancer. Although the MUIS-A has been adapted into several languages, its focus is primarily on disease uncertainty and does not address the specific uncertainties related to ongoing treatments such as dialysis. In contrast, the Uncertainty about Disease and Treatment Scale (UC-D&TS), designed by Rahimi Esbo et al. (2024), specifically targets the dual dimensions of disease and treatment-related uncertainty in hemodialysis patients. The uniqueness of this scale lies in its inclusion of treatment-specific factors, such as concerns about treatment efficacy, considerations of potential side effects, uncertainty regarding alternative treatment options, and doubts about the long-term outcomes of treatment. These factors are especially important for hemodialysis patients, who face a unique and ongoing treatment regimen. Evidence confirms the UC-D&TS as a valid and reliable tool quantifying uncertainty in HD patients, demonstrating associations with maladaptive coping (e.g., behavioral disengagement) and poor treatment adherence. This scale enables the systematic assessment of disease and treatment-related uncertainty, a critical psychological construct in chronic kidney disease management. Findings from the UC-D&TS can inform tailored care strategies, addressing unique psychosocial needs of hemodialysis patients (Payne and van den Akker, 2022). Given the importance and advantages of this scale, the reliability and validation of a verified Chinese version of this tool becomes imperative.

Psychological theory proposes that personality traits (e.g., extraversion, intelligence) function as latent variables (constructs), which resist direct observation but manifest through behavioral indicators—such as performance on standardized assessments—to infer an individual’s standing on specific latent dimensions. This operationalization defines the “reflective measurement model (Davoudi et al., 2023; Abdelrahman et al., 2025).” Although latent constructs provide the foundational theoretical framework for explicating observable behavioral variation and covariation, their quantification via behavioral metrics raises profound questions regarding inherent quantitative structural properties, particularly measurement invariance and sensitivity to dynamic intra-construct fluctuations. Conventional psychometric methodologies, exemplified by exploratory factor analysis, remain widely utilized; however, they frequently fail to accommodate the intricate and dynamic interdependencies between psychological constructs and their observable manifestations. Given that Mishel’s Uncertainty in Illness Theory conceptualizes uncertainty as a dynamic cognitive process in which its components mutually influence one another, A network framework aligns more closely with this theoretical view by modeling uncertainty as an interconnected system, allowing central nodes to represent the most influential cognitive elements within this processm, incorporating topological features such as nodal positioning, structural architecture, connection topology, and dyadic properties (Hevey, 2018). Consistent with Mishel’s conceptualization of uncertainty as a dynamic cognitive process, the network perspective allows observed components to be treated as mutually interacting elements rather than solely as reflective indicators of a latent construct. Within this domain, Exploratory Graph Analysis (EGA) constitutes a significant and innovative branch. Diverging from model-driven paradigms, EGA implements a data-driven framework to construct relational graphs—where nodes represent variables and edges denote statistical associations—algorithmically identifying core structural elements (e.g., central nodes, cluster communities, connectivity configurations). This approach reveals latent associative patterns elusive to traditional techniques, yielding enhanced intuitive insights into data architecture (Golino et al., 2020; Golino and Demetriou, 2017).

This study aimed to translate and culturally adapt the UC-D&TS, systematically evaluate its reliability and validity among Chinese hemodialysis patients, and use network analysis to explore the latent structural relationships among uncertainty constructs, providing tools and theoretical support for precise identification and intervention in the future.

## 2 Materials and methods

### 2.1 Design and participants

A cross-sectional survey was conducted from March to June 2025 among patients undergoing hemodialysis at five tertiary A-grade hospitals located in the eastern and northeastern regions of China. A convenience sampling method was used. According to empirical rules, exploratory factor analysis requires each item to match 5∼10 sample cases. The UC-D&TS questionnaire contains 17 items. Considering 10% invalid questionnaires, the formula *n* = [17 × (10∼20)]/(1–10%) calculates the number to be included as 189∼378. Confirmatory factor analysis requires a minimum sample size of 200 cases (MacCallum et al., 2001). A sample size of 50 is a minimum one required for examining the test-retest reliability of a scale (De Vet et al., 2011). A total of 411 questionnaires were distributed, and 404 valid responses were collected, resulting in a valid response rate of 98.3%.

All participants provided written informed consent prior to enrollment. The researchers explained the purpose and procedures of the study to each patient before administering the questionnaire. Questionnaires were distributed and completed individually under the supervision of the research team. Participants were encouraged to respond honestly. Questionnaires that were incomplete or exhibited obvious logical inconsistencies in responses to the Uncertainty about Disease and Treatment Scale were excluded from the analysis. The survey was anonymous, except for a subsample of 50 patients who were asked to provide their medical record numbers for test–retest reliability assessment. Two weeks later, these 50 patients were re-contacted to complete the scale again. All participants were native Mandarin speakers.

### 2.2 Translation process

We obtained permission from Professor Zahra Fotokian to translate and verify the Chinese version of UC-D&TS. We followed the systematic flow of Brislin’s translation (Golino, 2001). The UC-D&TS was independently translated into Chinese by two medical professors who are proficient in English. Then, together with the researchers, they compared the two Chinese versions of the questionnaires they had translated, discussed and corrected the inconsistencies, and obtained the first draft of the Chinese version. According to Brislin’s translation-back translation method, two English experts who had not been exposed to the scale translated back the Chinese version of the first draft. Finally, the original scale, the first Chinese draft, and the back-translated English version were compared and discussed by a psychologist and an expert familiar with Chinese and Western nursing culture to ensure semantic, conceptual, and standard equivalence. A pilot study was carried out among 10 patients undergoing hemodialysis. They completed the scale and were then asked about their understanding of the introduction, items, and response options. Items 5, 13, 17 were revised based on respondent feedback, literature review, and group discussion (details in Supplementary Appendix 1).

### 2.3 Measurements

All participants completed the UC-D&TS, the MUIS-A. Furthermore, participants were also asked to complete a checklist assessing sociodemographic variables (e.g., sex, age, marital status, degree of education) and clinical variables (e.g., duration of dialysis, complications, Renal transplant experience).

#### 2.3.1 Uncertainty about Disease and Treatment Scale

The UC-D&TS consists of 17 items, including 3 items assess self-uncertainty, 4 items assess uncertain situation, 3 items assess uncertain future, 4 items assess uncertainty of treatment outcomes, and 3 items assess information uncertainty. The items were rated on a 5-point Likert scale (strongly agree = 5, somewhat agree = 4, neither agree nor disagree = 3, somewhat disagree = 2, disagree = l), with the total score ranging strongly between 17 and 85. A lower score indicates less uncertainty, while a higher score suggests more uncertainty. The S-CVI/Ave was 0.98, and the Cronbach’s α coefficient of the scale was 0.828.

#### 2.3.2 Mishel Uncertainty In Illness Scale–adult form

The MUIS-A was developed by Mishel (1981) in 1981 to measure the level of illness-related uncertainty in adult patients. In 1997, Chinese scholar Shu and Xiu (1996) translated and revised the scale for use in China, and conducted reliability and validity testing. The scale consists of two dimensions—ambiguity and complexity—and includes a total of 25 items. The Cronbach’s alpha coefficient is 0.865, and the content validity index is 0.92, indicating good reliability and validity. As a measurement tool developed based on the actual conditions of adult patients in China, the MUIS-A effectively captures the degree of uncertainty patients experience during the course of illness progression. Given its similarity to the concept of disease and treatment uncertainty examined in this study, and its status as the most widely used illness uncertainty scale in China, the MUIS-A is adopted in this study as a criterion-related validity measure.

### 2.4 Statistical analysis of data

Data were analyzed using IBM SPSS 27.0 for item analysis, reliability analysis, and exploratory factor analysis (EFA); AMOS 28.0 for confirmatory factor analysis (CFA); and R software (version 4.5.0, GUI 1.81 Big Sur ARM build) with RStudio (version 2025.05.1 + 513) for network analyses, including EGA and bootEGA implemented via the qgraph and EGAnet packages.

#### 2.4.1 Item analysis

The critical ratio method was used to test the discrimination of each item in the scale, and the correlation coefficient method was used to test the representativeness of each item in the scale. The total score for each scale was ranked in ascending order, and the top 27% (high group) and bottom 27% (low group) were compared using an independent samples *t*-test to assess item discrimination. A critical ratio (CR) ≥ 3 with *p* < 0.05 was considered indicative of acceptable item discrimination. Item homogeneity was assessed by calculating the correlation coefficient(r) between each item and the total scale score, values ≥ 0.4 suggested good internal consistency (Raykov and Marcoulides, 2016).

#### 2.4.2 Reliability analysis

Internal consistency of the Chinese version of the scale was evaluated using Cronbach’s alpha, McDonald’s omega (McDonald and Roderick, 1999), and test-retest reliability. For test-retest analysis, approximately 50 participants were assessed at a 2-week interval (Lu et al., 2022). Test–retest reliability was assessed using a two-way random-effects model with absolute agreement for single measurements [ICC(2,1)]. Reliability was deemed acceptable when Cronbach’s α, McDonald’s omega, and test-retest reliability all exceeded 0.70 (Marques et al., 2021; Frost et al., 2007). Split-half reliability was calculated by dividing the scale into odd- and even-numbered items, then correlating the scores of the two halves and applying the Spearman-Brown formula for adjustment.

#### 2.4.3 Validity analysis

Content validity was evaluated by a panel of seven experts (psychologists and medical specialists) using the Delphi method. Experts rated each item on a 4-point Likert scale (1 = inappropriate to 4 = very appropriate). Item-level content validity index (I-CVI) was calculated by dividing the number of experts assigning a rating of 3 or 4 by the total number of experts. Scale-level content validity index (S-CVI) was the average of the I-CVIs across all items. Validity was considered adequate if I-CVI ≥ 0.78 and S-CVI ≥ 0.90 (Almanasreh et al., 2019).

To evaluate construct validity, exploratory factor analysis (EFA) was conducted using SPSS 27.0, and confirmatory factor analysis (CFA) was performed using AMOS 28.0. EFA was performed using Principal Axis Factoring (PAF) with Promax rotation, guided by the scree plot (Mabel and Olayemi, 2020). Sample adequacy was evaluated using the Kaiser-Meyer-Olkin (KMO) measure and Bartlett’s test of sphericity. A KMO value > 0.60 and *p* < 0.05 for Bartlett’s test were prerequisites for factor analysis (Tobias and Carlson, 1969), with values between 0.8 and 0.9 considered excellent. Additionally, parallel analysis was conducted to further validate the factor structure identified through exploratory factor analysis (EFA).

To analyze the structural validity of a scale, CFA is conducted using structural equation modeling (SEM) (Browne and Cudeck, 1992). The maximum likelihood method is applied for CFA. The following indices are used to evaluate model fit: the chi-square to degrees of freedom ratio (χ^2^/df), the root mean square error of approximation (RMSEA), the comparative fit index (CFI), the incremental fit index (IFI), the Tucker-Lewis index (TLI), and the normed fit index (NFI). A model is considered to have excellent fit if it meets the following criteria: χ^2^/df < 3.000, RMSEA < 0.080, IFI and NFI > 0.900, and TLI and CFI > 0.950 (Tobias and Carlson, 1969; Stegmann, 2017; F’an and Sivo, 2007).

Convergent and Discriminant validity was determined by calculating the Composite Reliability (CR) and Average Variance Extracted (AVE); CR > 0.70 and AVE > 0.50 were interpreted as evidence of good convergent validity (Kong et al., 2024; Yang et al., 2022). To evaluate the criterion validity of the scale, MUIS-A was selected as the external criterion, given its conceptual alignment with the current instrument. Both scales are designed to assess patients’ perceptions of uncertainty during the illness experience. Pearson correlation analysis was conducted to examine the degree of association between the two measures, thereby providing evidence for the criterion-related validity of the newly developed scale.

#### 2.4.4 Network analyses

Exploratory Graph Analysis (EGA) was performed using R software (version 4.5.0, GUI 1.81 Big Sur ARM build) and R studio (version2025.05.1 + 513). EGA integrates Gaussian graphical modeling via the qgraph package (Epskamp et al., 2012) with LASSO regularization and Extended Bayesian Information Criterion (EBIC) model selection (Golino and Epskamp, 2017; Golino et al., 2022). The resulting sparse inverse covariance matrix was used to estimate partial correlations, and the Walktrap community detection algorithm from the igraph package (Yan and Yao, 2015) was applied to detect highly connected subgraphs or communities.

To assess the stability of the dimensional structure, a bootstrap procedure with 1,000 resamples was conducted using the boot package (Canty and Ripley, 2020). This procedure involved re-sampling the data with replacement to evaluate the stability of the identified dimensional structure across multiple iterations. The bootstrapping procedure provided estimates for the variability in the number of dimensions and allowed for the evaluation of consistency in the factor structure across the samples.

Additionally, centrality indices such as expected influence and strength were calculated using the qgraph package. To assess the stability of these centrality indices, stability checks were conducted using the bootstrapping procedure from the boot package, where centrality indices were recalculated across 1,000 resamples to evaluate their robustness.

### 2.5 Ethical approval

The study protocol was approved by the Ethics Committee of the Naval Medical University (Approval No.2022BJZ09). All participants were fully informed about the study purpose and procedures and provided written informed consent prior to participation. The survey was conducted anonymously to protect participant privacy. For the subsample of 50 patients who participated in the test–retest reliability assessment, written informed consent was specifically obtained to collect their medical record numbers, which were used solely to match responses between the two assessments. These identifiers were removed immediately after matching, and the final dataset contained no personally identifiable information. All procedures complied with the ethical principles outlined in the 1964 Declaration of Helsinki and its subsequent amendments.

## 3 Results

### 3.1 Descriptive statistics

From the initially selected population-based sample, a total of 432 individuals were assessed for eligibility for data analysis. Among them, 17 participants were excluded due to incomplete responses to the UC-D&TS. An additional 11 participants were excluded due to abnormally short response times—defined as completing the questionnaire in under 5 min—which suggested potential random or inattentive answering. As a result, 404 participants were included in the final analytic sample. All samples were randomly divided into independent subgroups: Subgroup 1 was allocated for evaluating CFA (202 participants), while the subsequent subgroup was designated for evaluating EFA (202 participants).

In our study, the majority were male (67.6%). The mean age of the patients was 60 years (range: 18∼94 years), and the mean duration of hemodialysis was 4 years [(range: approximately 1 month (0.08 years)∼24 years)]. Nearly one third of the patients had hypertension as the primary disease. The characteristics of both subsamples were similar to those of the total sample, and no significant differences in characteristics were found between two subsamples. Demographic characteristics of the study sample are presented in Table 1.

**TABLE 1:** Demographic characteristics of participants.

Characteristics	Total (*N* = 404)	EFA (*N* = 202)	CFA (*N* = 202)
Age (years)	60.73 ± 14.66	57.19 ± 15.08	64.27 ± 13.35
**Sex**			
Male	67.6(273)	63.9(129)	71.3(144)
Female	32.4(131)	36.1(73)	28.7(58)
**Marital status**			
Married	84.2(340)	85.6(173)	82.7(167)
Unmarried	10.1(41)	9.9(20)	10.4(21)
Widowed spouse	4.5(18)	2.5(5)	6.4(13)
Get divorced	1.2(5)	2.0(4)	0.5(1)
**Education**			
Primary or lower	9.7(39)	11.4(23)	7.9(16)
Junior high	34.9(141)	38.1(77)	31.7(64)
Senior or vocational	34.2(138)	30.7(62)	37.6(76)
College or bachelor	20.5(83)	18.8(38)	22.3(45)
Postgraduate or higher	0.7(3)	1.0(2)	0.5(1)
**Payment methods for medical expenses**			
Employee health insurance	73.0(295)	73.3(148)	72.8(147)
Medical insurance for residents	20.8(84)	18.3(37)	23.3(47)
Rural cooperative medical insurance	1.7(7)	3.0(6)	0.5(1)
Business insurance	2.7(11)	4.0(8)	1.5(3)
All at your own expense	1.7(7)	1.5(3)	2.0(4)
Duration of dialysis (years)	4.87 ± 4.03	5.52 ± 4.11	4.22 ± 3.85
**Dialysis-related complications**			
Yes	59.2(239)	74.3(150)	44.1(89)
No	40.8(165)	25.7(52)	55.9(11.3)
**Renal transplant experience**			
Yes	5.0(20)	3.5(7)	6.4(13)
No	95.0(384)	96.5(195)	93.6(189)
**Renal transplant willingness**			
None	76.5(309)	74.3(150)	78.7(159)
Moderate	11.9(48)	10.4(21)	13.4(27)
Strong	11.6(47)	15.3(31)	7.9(16)

### 3.2 Item analysis and ceiling and floor effects

The Chinese version of UC-D&TS consists of 17 items (without the items for clinical significance). The range of the critical ratio (CR) for all items was between –9.617 and –25.461, while the correlation coefficient (r) between each item and the overall score ranged from 0.513 to 0.759. Additionally, all items’ mean and standard deviation (SD) values ranged from 2.267 to 3.210 and 0.959 to 1.266, respectively. Details are shown in Table 2.

**TABLE 2:** *T*-test results of the samples and correlation coefficient of measured concept.

Item	Mean	SD	CR	*r*
1. I am not certain about my ability to perform my daily activities considering the type of treatment I am receiving for my disease.	2.639	1.100	–10.760**	0.513**
2. I am not certain if I can effectively utilize strategies to deal with the side effects associatedwith my treatment.	3.082	1.076	–25.247**	0.737**
3. I am not certain of my ability to be prepared to handle crisis situations related to hemodialysis.	3.210	1.106	–23.647**	0.727**
4. I am not certain if I can accept other treatment methods for my disease.	2.946	0.959	–18.348**	0.657**
5. At times, when I become exhausted from the conditions associated with hemodialysis, I am unsure about continuing hemodialysis treatment.	2.267	1.065	–9.617**	0.533**
6. I am not certain that I can pursue my life goals and aspirations based on the type of treatment I am receiving.	3.203	1.100	–20.292**	0.693**
7. I am not certain about having a favorable social or family status in the future due to my disease or hemodialysis.	3.101	1.128	–16.853**	0.679**
8. I am in a state where nothing in my life can be relied upon with certainty.	2.438	1.187	–12.445**	0.580**
9. I am not certain of what not to do in the future for my treatment.	2.592	1.230	–21.766**	0.751**
10. Whenever an issue arisesing my treatment, I find it difficult to make a decision about it easily.	2.985	1.096	–25.461**	0.740**
11. I have many unanswered questions about the future of my treatment.	2.599	1.227	–21.695**	0.759**
12. I am not certain whether hemodialysis is a suitable approach for extending mylifespan.	3.119	1.266	–22.015**	0.735**
13. I am not certain whether training the treatment team can reduce the complications of hemodialysis.	3.082	0.961	–14.229**	0.631**
14. I am not certain whether adhering to treatment will result in a longer lifespan.	3.079	1.242	–23.155**	0.736**
15. I am not certain whether I have enough information about kidney transplantation or not.	3.101	0.980	–18.179**	0.644**
16. I am not certain if a kidney transplant is better than hemodialysis.	3.000	0.984	–18.117**	0.638**
17. I am not very certain about the information I have regarding my arteriovenous fistula or central venous catheter.	2.525	1.101	–17.667**	0.693**

**p < 0.01.

Calculate the proportion of samples with the lowest (highest) scores relative to the total sample size to identify floor and ceiling effects for each scale item. A proportion exceeding 15% indicates significant floor (ceiling) effects. No patient achieved the minimum score of 17 or the maximum score of 85, indicating the absence of floor or ceiling effects for the overall scale. The proportion of patients scoring the maximum on any single item was below 15%, suggesting no ceiling effect. However, for item 4, 7, 8, and 9, the proportion of patients scoring the minimum exceeded 15%, indicating the presence of a floor effect. Details are shown in Table 3.

**TABLE 3:** Ceiling and floor effects.

Item	The lowest score n (percentage, %)	The highest score n (percentage, %)
Total score	0(0.0%)	0(0.0%)
1. I am not certain about my ability to perform my daily activities considering the type of treatment I am receiving for my disease.	41(10.2%)	19(4.7%)
2. I am not certain if I can effectively utilize strategies to deal with the side effects associated with my treatment.	10(2.5%)	32(8.0%)
3. I am not certain of my ability to be prepared to handle crisis situations related to hemodialysis.	4(1.0%)	54(13.4%)
4. I am not certain if I can accept other treatment methods for my disease.	17(4.2%)	5(1.2%)
5. At times, when I become exhausted from the conditions associated with hemodialysis, I am unsure about continuing hemodialysis treatment.	97(24.0%)	7(1.7%)
6. I am not certain that I can pursue my life goals and aspirations based on the type of treatment I am receiving.	17(4.2%)	40(10.0%)
7. I am not certain about having a favorable social or family status in the future due to my disease or hemodialysis.	29(7.2%)	41(10.2%)
8. I am in a state where nothing in my life can be relied upon with certainty.	99(24.5%)	18(4.5%)
9. I am not certain of what not to do in the future for my treatment.	83(20.5%)	19(4.7%)
10. Whenever an issue arisesing my treatment, I find it difficult to make a decision about it easily.	13(3.2%)	38(9.4%)
11. I have many unanswered questions about the future of my treatment.	82(20.3%)	25(6.2%)
12. I am not certain whether hemodialysis is a suitable approach for extending mylifespan.	44(10.9%)	48(11.9%)
13. I am not certain whether training the treatment team can reduce the complications of hemodialysis.	5(1.2%)	27(6.7%)
14. I am not certain whether adhering to treatment will result in a longer lifespan.	46(11.4%)	36(8.9%)
15. I am not certain whether I have enough information about kidney transplantation or not.	23(5.7%)	7(1.7%)
16. I am not certain if a kidney transplant is better than hemodialysis.	23(5.7%)	6(1.5%)
17. I am not very certain about the information I have regarding my arteriovenous fistula or central venous catheter.	50(12.4%)	24(5.9%)

### 3.3 Content validity

The content validity index for each item ranges from 0.857 to 1.000, and the content validity index for the overall scale is 0.933, indicating strong content validity.

### 3.4 Exploratory factor analysis

**FIGURE 1:**
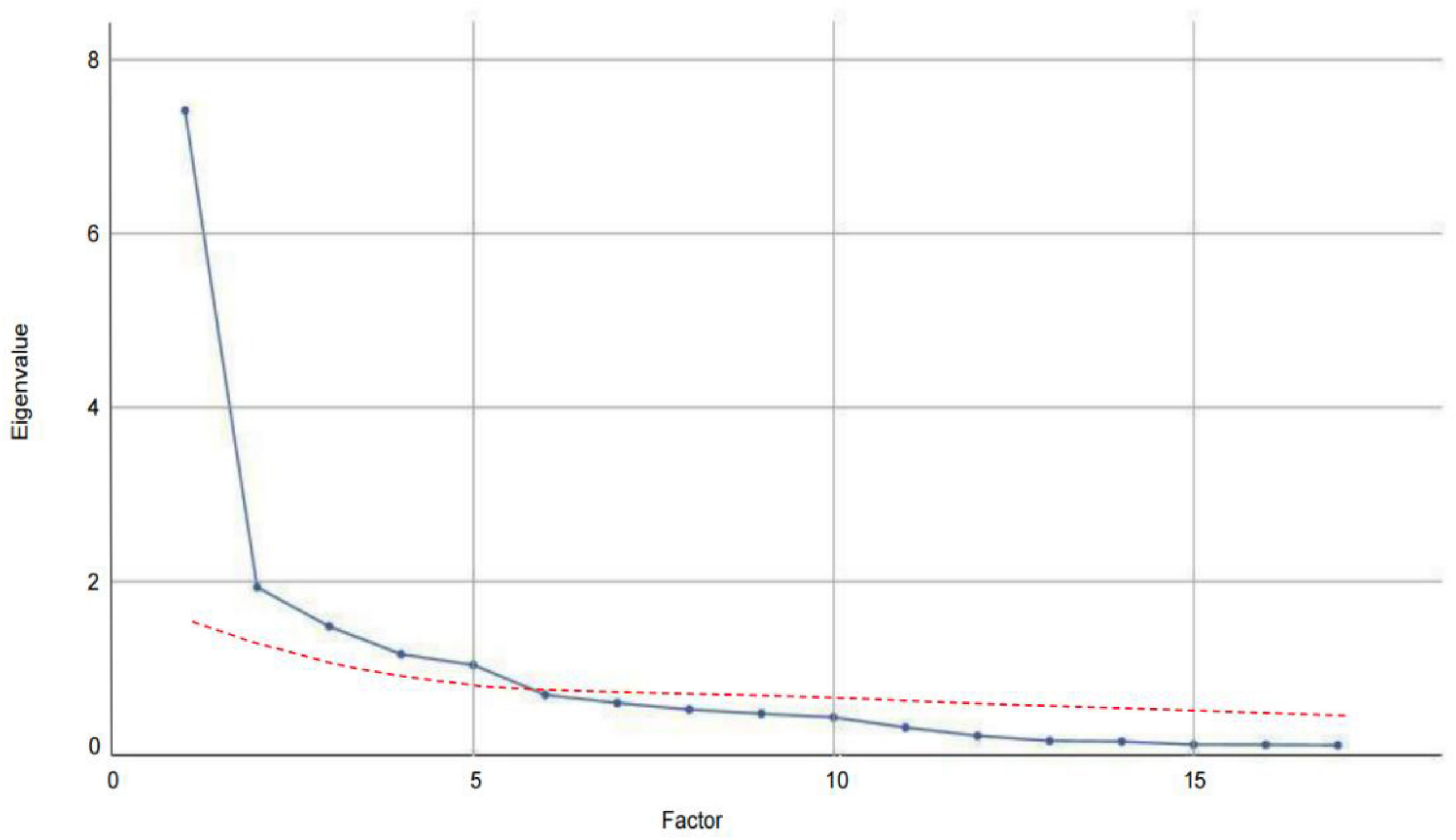
Comparison of eigenvalues for real and random data in parallel analysis.

KMO measure of sampling adequacy yielded a strong value of 0.847, indicating that the data were well-suited for exploratory factor analysis. Additionally, Bartlett’s test of sphericity was statistically significant (χ^2^ = 2271.505, *p* < 0.001), further confirming the appropriateness of the dataset for factor analysis and reinforcing confidence in the subsequent procedures. An examination of the factor structure of the Chinese version of the UC-D&TS revealed five distinct factors with eigenvalues greater than one, as supported by the scree plot (Figure 1, blue line). These factors made substantial contributions to the explained variance of the UC- D&TS construct. Using Promax rotation, the analysis demonstrated that these five factors collectively accounted for 72.99% of the total variance (Table 4). This comprehensive factor analysis elucidates the underlying dimensions of the scale and offers valuable insights into its structural composition, thereby enhancing our understanding of reinforcement sensitivity.

**TABLE 4:** Exploratory factor analysis for Chinese version of UC-D&TS.

Item	Factor 1	Factor 2	Factor 3	Factor 4	Factor 5
9. I am not certain of what not to do in the future for my treatment.	0.842	0.174	0.113	0.252	0.168
11. I have many unanswered questions about the future of my treatment.	0.835	0.204	0.115	0.234	0.227
17. I am not very certain about the information I have regarding my arteriovenous fistula or central venous catheter.	0.715	0.158	0.383	0.042	0.246
1. I am not certain about my ability to perform my daily activities considering the type of treatment I am receiving for my disease.	0.399	0.220	0.031	0.063	0.048
5. At times, when I become exhausted from the conditions associated with hemodialysis, I am unsure about continuing hemodialysis treatment.	0.315	0.006	0.249	0.121	0.208
3. I am not certain of my ability to be prepared to handle crisis situations related to hemodialysis.	0.148	0.879	0.143	0.245	0.174
10. Whenever an issue arises regarding my treatment, I find it difficult to make a decision about it easily.	0.284	0.834	0.137	0.188	0.171
2. I am not certain if I can effectively utilize strategies to deal with the side effects associated with my treatment.	0.249	0.822	0.149	0.242	0.145
16. I am not certain if a kidney transplant is better than hemodialysis.	0.079	0.095	0.919	0.121	0.014
15. I am not certain whether I have enough information about kidney transplantation or not.	0.177	0.140	0.899	0.038	0.028
4. I am not certain if I can accept other treatment methods for my disease.	0.078	0.135	0.687	0.258	0.194
12. I am not certain whether hemodialysis is a suitable approach for extending my lifespan.	0.245	0.169	0.120	0.846	0.197
14. I am not certain whether adhering to treatment will result in a longer lifespan.	0.227	0.216	0.184	0.823	0.158
13. I am not certain whether training the treatment team can reduce the complications of hemodialysis.	0.100	0.267	0.081	0.694	0.201
6. I am not certain that I can pursue my life goals and aspirations based on the type of treatment I am receiving.	0.150	0.195	0.091	0.294	0.818
7. I am not certain about having a favorable social or family status in the future due to my disease or hemodialysis.	0.292	0.140	0.003	0.229	0.808
8. I am in a state where nothing in my life can be relied upon with certainty.	0.163	0.129	0.105	0.061	0.769

Two items (Item 1 and Item 5) were excluded from the factor structure due to low factor loadings (< 0.40) across all extracted components in the exploratory factor analysis. These items failed to meet the loading criteria for inclusion in any specific factor, indicating a weak association with the underlying constructs.

The first factor, “Coping and Decision-making Uncertainty,” consisting of three items, accounted for 15.53% of the total variance (eigenvalue = 2.64). The second factor, “Outcome Expectancy Uncertainty,” comprising three items, explained 15.04% of the total variance (eigenvalue = 2.56). The third factor, “Treatment-related Knowledge Uncertainty,” consisting of three items, accounted for 14.68% of the variance (eigenvalue = 2.50). The fourth factor, “Alternative Treatment Uncertainty,” composed of three items, explained 14.08% of the variance (eigenvalue = 2.39). The fifth factor, “Existential and Social Role Uncertainty,” comprising three items, accounted for 13.66% of the variance (eigenvalue = 2.32).

Additionally, to validate the factor structure extracted through exploratory factor analysis, parallel analysis was conducted, with the results presented in Figure 1. The actual eigenvalues (blue line) represent those derived from the real data of the UC- D&TS scale, while the random eigenvalues (red line) reflect the average values obtained from 100 iterations of randomly simulated datasets. These random eigenvalues served as a benchmark for determining the number of meaningful factors to retain. As illustrated in Figure 1, the actual eigenvalues for the first five factors clearly exceed the corresponding random eigenvalues, thereby confirming that five factors are statistically significant. This alignment between the empirical data and the simulation results provides strong support for the five-factor solution.

### 3.5 Exploratory graph analysis

**FIGURE 2:**
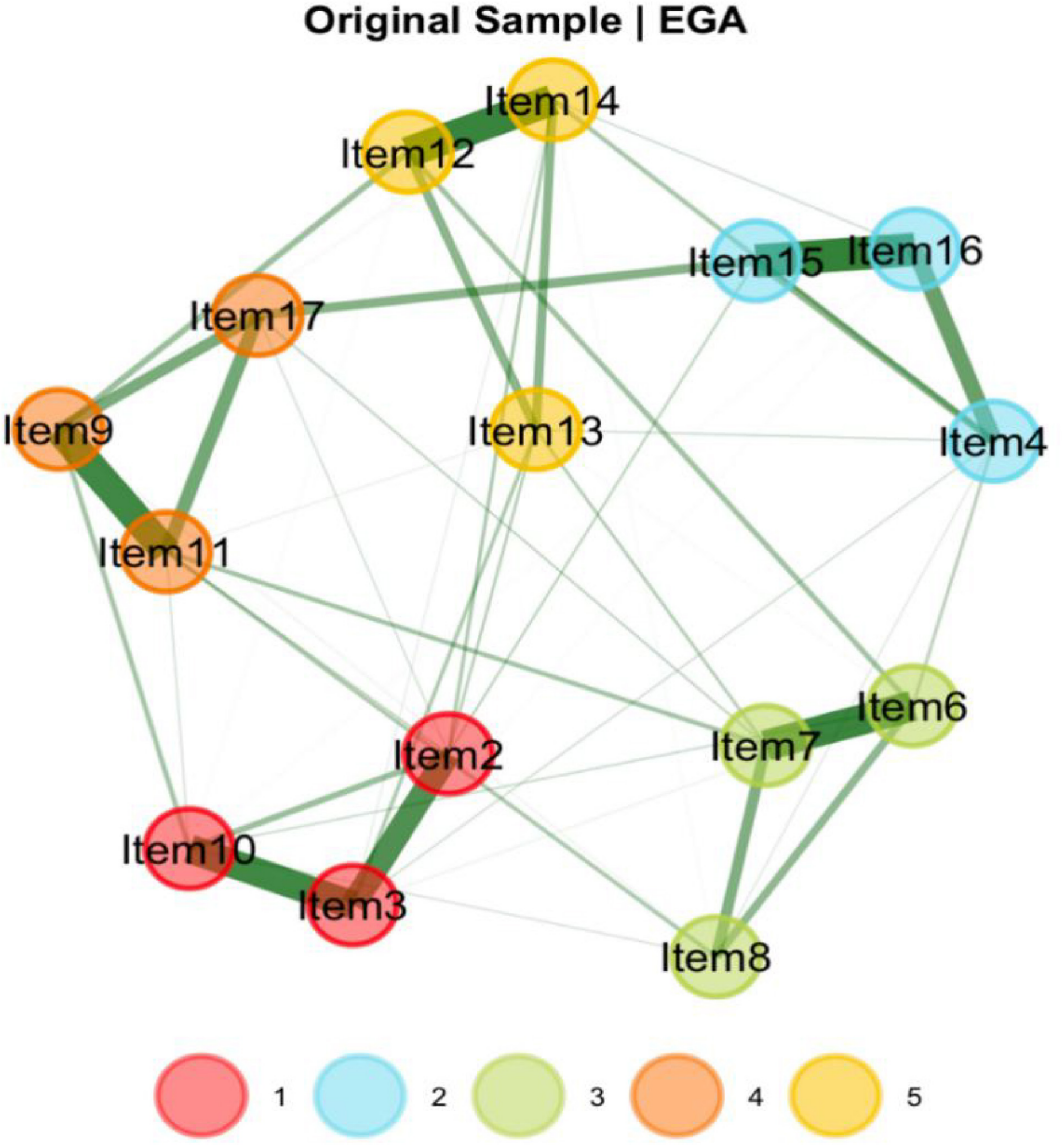
Dimensionality results for EGA for the UC-D&Ts.

EGA identified a five-dimensional structure for the Uncertainty about Disease and Treatment Scale (Figure 2). As illustrated in Figure 2, the item groupings closely correspond to the factor structure revealed by the EFA. The analysis employed a graphical LASSO (Glasso) model in conjunction with the walktrap community detection algorithm, while unidimensionality was assessed using the leading eigenvalue method.

To evaluate the stability of the dimensional structure, a bootstrap procedure with 1,000 resamples was performed. The results indicated a median of five dimensions across the bootstrap samples, with a standard error of approximately 0.05. The 95% confidence interval for the number of dimensions ranged from 4.92 to 5.08.

**FIGURE 3:**
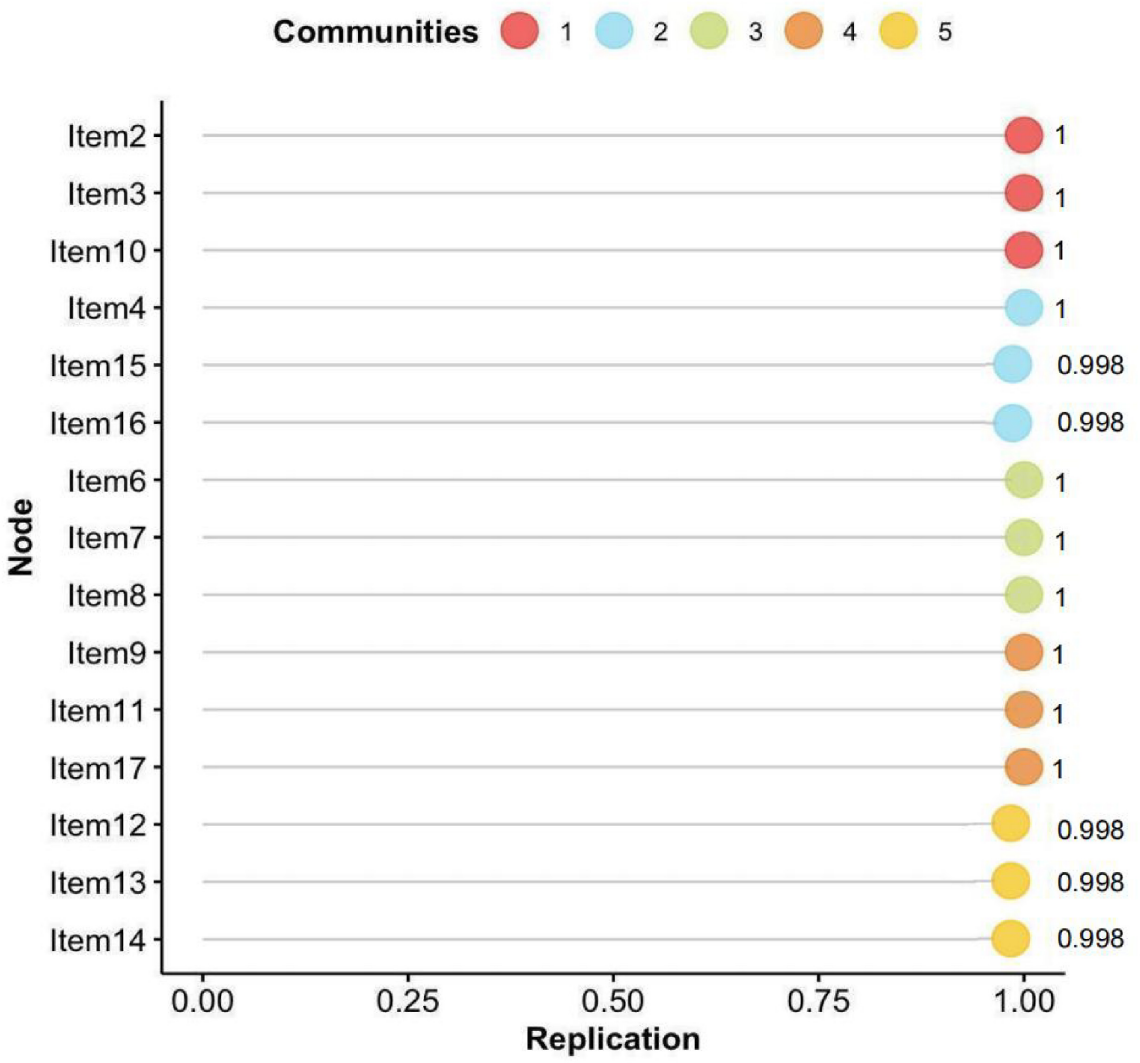
Probability distribution of each symptom’s association with the community in which it was first identified by EGA, based on 1,000 bootstrap iterations.

The bootstrapped EGA (bootEGA) further confirmed the robustness of the five-dimensional solution, which was replicated in 998 out of 1,000 iterations—suggesting that this structure is both dominant and highly stable. Figure 3 illustrates the likelihood of each item consistently clustering within its originally identified dimension throughout the 1,000 bootstrap iterations. The vast majority of items showed replication frequencies exceeding 0.998, indicating a high degree of structural consistency.

**FIGURE 4:**
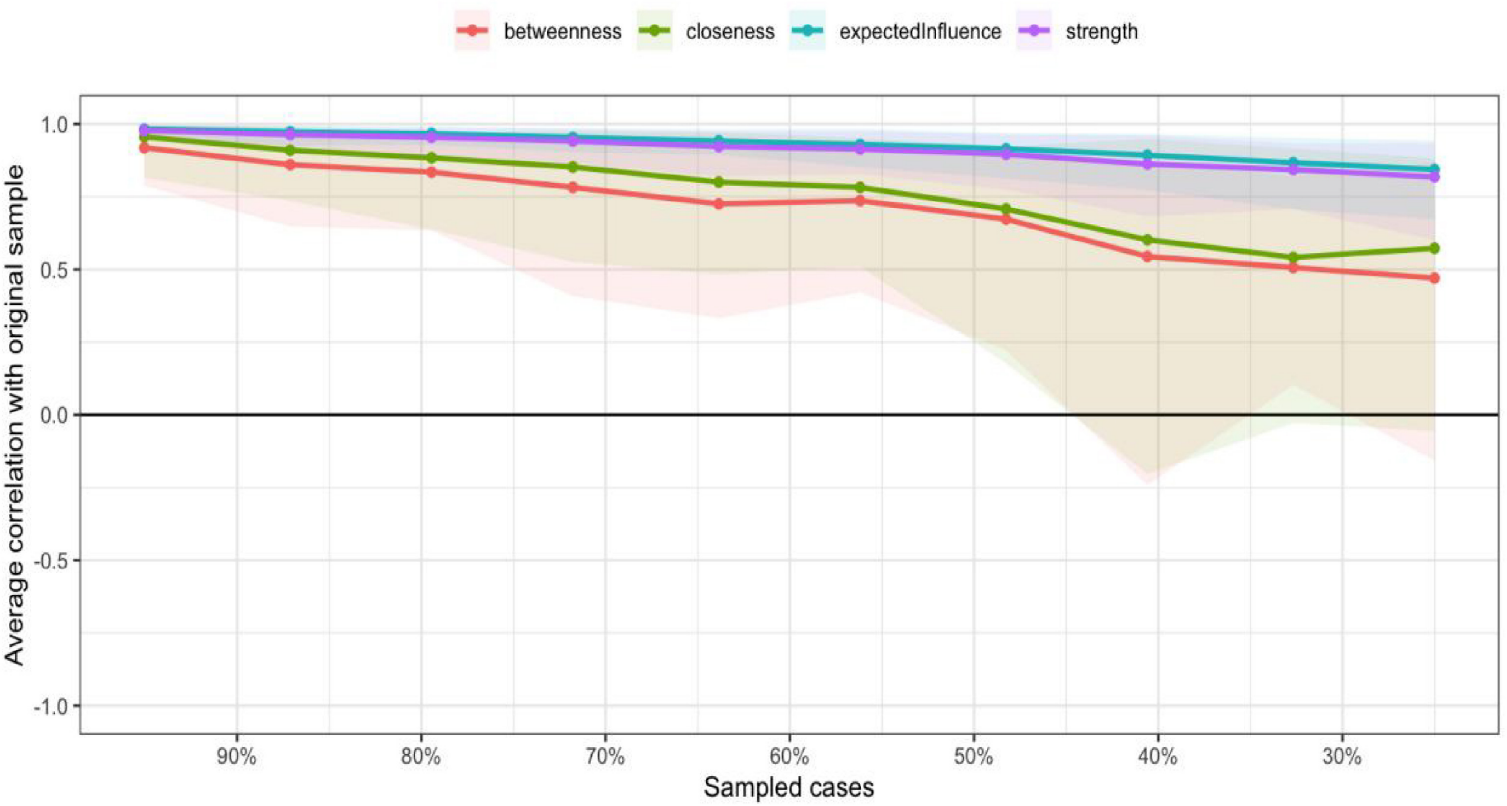
The centrality estimates for the items of the UC-D&Ts.

**FIGURE 5:**
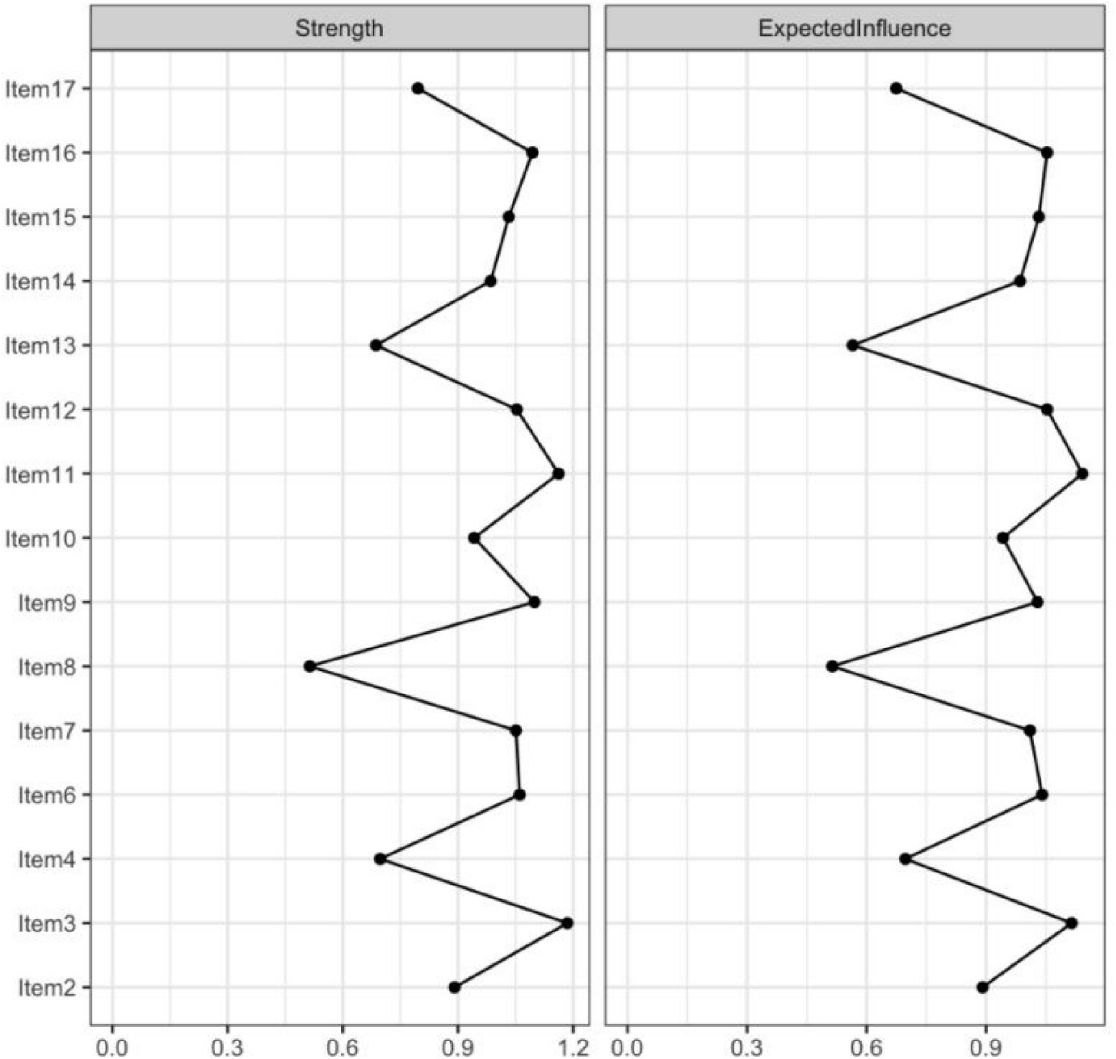
The centrality estimates for the items of the UC-D&Ts.

In the current study, centrality indices were examined to identify the most influential symptoms in the network (Figure 4). Among these indices, expected influence and strength were emphasized due to their high stability across bootstrap samples (CS-coefficients of 0.75 and 0.67, respectively), supporting the robustness of the centrality findings. Building upon this, the network analysis revealed that Items 11 (“I have many unanswered questions about the future of my treatment.”), 3 (“I am not certain of my ability to be prepared to handle crisis situations related to hemodialysis”), 16 (“I am not certain if a kidney transplant is better than hemodialysis”), and 12 (“I am not certain whether hemodialysis is a suitable approach for extending mylifespan”) exhibited the highest expected influence and strength values. These results highlight their pivotal roles as core components within patients’ uncertainty about disease and treatment, reflecting key areas of cognitive doubt and suggesting that clinical interventions should prioritize addressing these concerns to alleviate psychological distress and promote adaptive coping (Figure 5).

### 3.6 Confirmatory factor analysis

**FIGURE 6:**
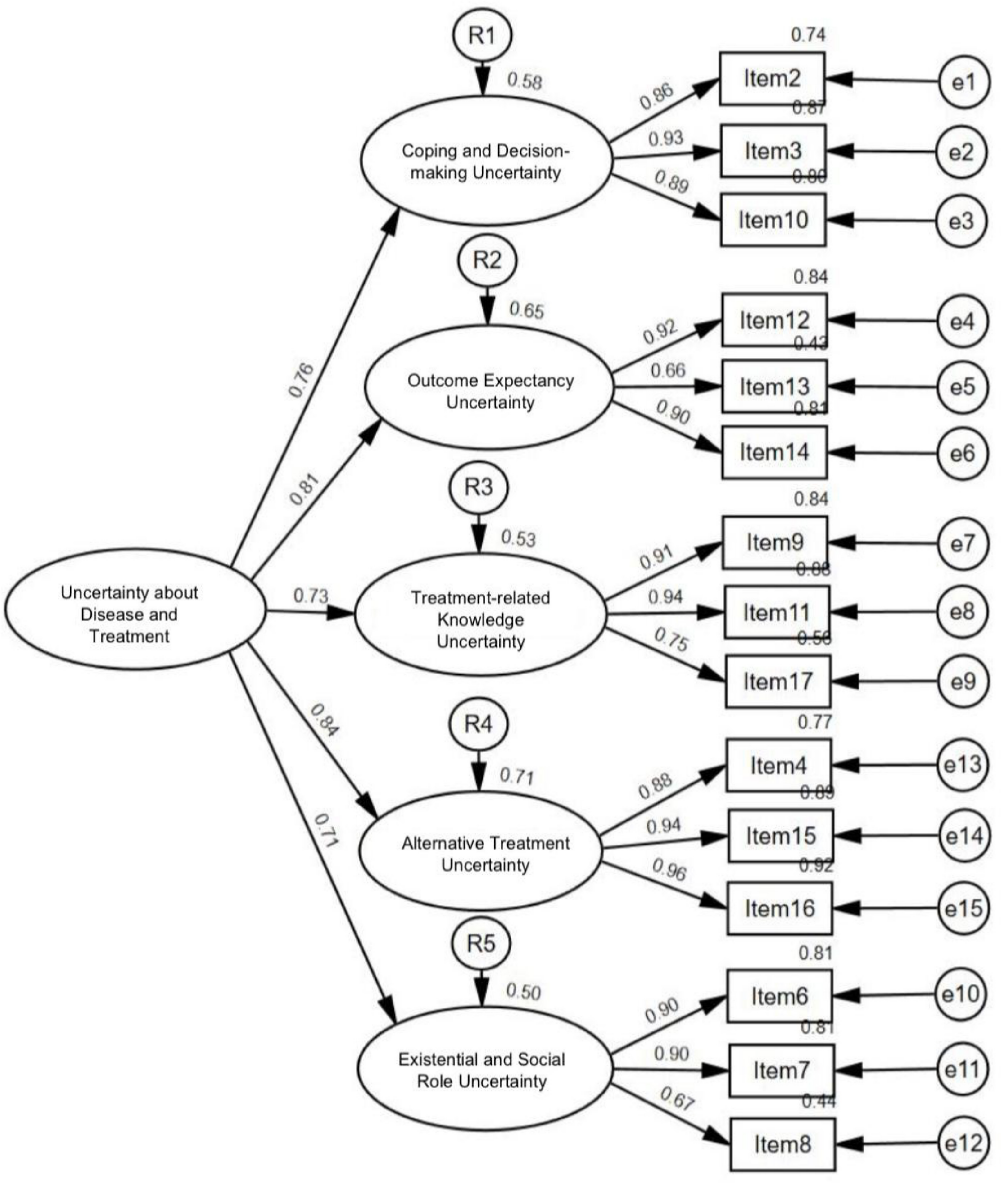
Final measurement model of uncertainty about disease and treatment from second-order confirmatory factor analysis.

A second-order CFA was conducted to examine the hierarchical relationships among constructs by specifying a second-order latent factor with the five first-order factors derived from EFA with subsample 1 (Figure 6). The results showed that the model fit indices were χ^2^(202) = 188.545, *p* < 0.001, RMSEA = 0.077 with the 90% confidence interval 0.063–0.093, SRMR = 0.049, NFI = 0.931, IFI = 0.961, TLI = 0.951, CFI = 0.961, indicating an acceptable fit to the data. All 15 items were significantly loaded onto their respective first-order factors with standardized loadings, ranging from 0.66 to 0.96. For the second-order latent factor, the five standardized second-order factor loadings were statistically significant, ranging from 0.71 to 0.84 (Table 5).

**TABLE 5:** Fit indices of the proposed model.

Model	χ^2^	df	χ^2^/df	NFI	IFI	TLI	CFI	RMSEA [90% CI]	SRMR
First oder	179.397	80	2.242	0.934	0.962	0.950	0.962	0.079 [0.063, 0.094]	0.044
Second order	188.545	85	2.218	0.931	0.961	0.951	0.961	0.077 [0.063, 0.093]	0.049

χ^2^, Chi-square; df, Degrees of freedom; χ^2^/df, Chi-square to degrees of freedom ratio; NFI, Normed Fit Index; IFI, Incremental Fit Index; TLI, Tucker-Lewis Index; CFI, Comparative Fit Index; RMSEA, Root Mean Square Error of Approximation; SRMR, Standardized Root Mean Square Residual.

### 3.7 Convergent and discriminant validity

The scale demonstrated acceptable convergent validity according to the Fornell and Larcker criterion, as all factors showed AVE values above 0.50 (range from 0.690 to 0.857) and composite reliability (CR) values above 0.70 (range from 0.868 to 0.947), with each CR exceeding its corresponding AVE. Discriminant validity was also supported. The square root of the AVE for each latent construct was greater than its correlations with all other constructs, indicating satisfactory discriminant validity.

### 3.8 Criterion validity

Results showed the correlations between the five dimensions of the UC- D&Ts and the Uncertainty subscale of the MUIS-A ranged from 0.487 to 0.556, while correlations with the Complexity subscale ranged from 0.506 to 0.609 (*P* < 0.001). These findings indicate that the UC- D&Ts is moderately and positively associated with related constructs measured by the MUIS-A, supporting its criterion-related validity (Table 6).

**TABLE 6:** Correlation between UC- D&Ts and MUIS-A scale.

	Coping and decision-making uncertainty	Outcome expectancy uncertainty	Treatment-related knowledge uncertainty	Altemative treatment uncertainty	Existential and social role uncertainty
	UC- D&Ts				
	1	2	3	4	5
**MUIS-A**					
Uncertainty	0.487**	0.535**	0.498**	0.553**	0.556**
Complexity	0.535**	0.539**	0.507**	0.609**	0.506**

**Significant on 0.01 level.

### 3.9 Reliability

The Cronbach’s alpha value for this scale was 0.925, the bootstrap CI for Cronbach’s α: [0.914, 0.934]. The five-dimensional Cronbach’s α values for this scale range from 0.844 to 0.924. The reliability analysis of the translated scale indicated good internal consistency, with Cronbach’s alpha values of 0.874 for the first half and 0.861 for the second half. The split-half reliability coefficient was 0.894. The item−to−total correlations ranged between 0.509 and 0.708. Therefore, the translated scale had suitable reliability. Also, McDonald’s ω coefficient for the total scale was 0.98, and those for the five dimensions ranged from 0.93 to 0.96 (Table 7).

**TABLE 7:** Reliability of the scale (*N* = 404).

Total/subdimension	Corrected item-total correlation coefficient	Cronbach’s alpha	McDonald’s ω coefficient
Total	–	0.925	0.98
Coping and decision-making uncertainty	0.684∼0.699	0.924	0.95
Outcome expectancy uncertainty	0.587∼0.686	0.854	0.96
Treatment-related knowledge uncertainty	0.642∼0.708	0.903	0.95
Alternative treatment uncertainty	0.596∼0.610	0.898	0.93
Existential and social role uncertainty	0.509∼0.649	0.844	0.93

In addition, 2 weeks after the initial survey, a random sample of 50 patients who had participated in the first round of data collection were selected for retesting using the same scale. The intra-class correlation coefficients (ICCs) for the retest ranged from 0.952 to 0.987, indicating excellent test-retest reliability.

## 4 Discussion

This study aimed to translate, culturally adapt, and psychometrically validate the UC-D&TS among patients undergoing hemodialysis in China. To our knowledge, this is the first study to introduce this tool to a Chinese-speaking population and to explore its psychometric properties through both classical test theory and modern psychometric techniques, including network analysis. The findings provide robust evidence supporting the reliability, validity, and structural stability of the Chinese version of the UC-D&TS, and further extend our understanding of uncertainty constructs in the hemodialysis context.

### 4.1 Principal findings and comparison with previous research

The translated UC-D&TS demonstrated excellent internal consistency, with a Cronbach’s alpha of 0.925 and McDonald’s omega of 0.980 for the total scale. The five extracted dimensions—Coping and Decision-Making Uncertainty, Outcome Expectancy Uncertainty, Treatment-Related Knowledge Uncertainty, Alternative Treatment Uncertainty, and Existential and Social Role Uncertainty—collectively accounted for approximately 73% of the total variance. These five factors were validated both by CFA and EGA, the latter of which further confirmed the scale’s structural integrity through bootstrapping, with over 99% dimensional replication across iterations. In addition, the scale demonstrated strong convergent and discriminant validity and moderate criterion-related validity with the MUIS-A, confirming its alignment with existing constructs while capturing unique features of uncertainty in HD populations.

During scale refinement, two items (Item 1 and Item 5) were removed due to consistently low factor loadings (< 0.40) across all components, which helped enhance the overall conceptual clarity and construct validity of the final scale. Item 1, which assessed general functional ability (“I am not certain about my ability to perform my daily activities…”), may not reflect treatment-specific uncertainty but rather general physical capacity or health status. In clinical practice, it is not uncommon for patients with significant psychological uncertainty to still demonstrate intact physical functioning, while older adults with physical limitations may not necessarily experience—or endorse—a strong sense of uncertainty. Furthermore, many patients do not attribute their reduced daily functioning directly to hemodialysis, suggesting that functional limitations may be conceptually distinct from treatment-related uncertainty. Accordingly, Item 1 was removed to maintain alignment with the cognitive-emotional focus of the scale. Item 5 (“At times, when I become exhausted from the conditions associated with hemodialysis, I am unsure about continuing with it.”) was excluded based on both psychometric and cultural grounds. Psychometrically, the item’s emphasis on emotional exhaustion and behavioral hesitation contrasts with the predominantly cognitive orientation of the other items, potentially reducing its discriminant validity within the factor structure. Culturally, the item may not resonate with core Chinese values rooted in Confucian ideals, which emphasize endurance, filial piety, and a strong sense of familial responsibility (Fan, 2011; Badanta et al., 2022). Under the influence of these values—particularly the belief in “doing one’s best and leaving the rest to fate”—many patients may experience a psychological burden against giving up treatment. As a result, they may feel a moral obligation to pursue life-sustaining therapies despite internal doubts, and may be reluctant to express uncertainty about continuing treatment, even in self-report. This cultural incongruence may have contributed to restricted response variability and weak factor loadings, leading to the item’s exclusion.

The UC-D&TS showed moderate correlations with the MUIS-A’s Uncertainty and Complexity dimensions, supporting its criterion-related validity while also indicating conceptual distinctions. While the MUIS-A assesses general illness-related uncertainty using two broad dimensions, the UC-D&TS was developed specifically for hemodialysis patients in China, capturing five more differentiated domains: self-capacity and preparedness, outcome expectations, informational mastery, decision-making, and role/life meaning.

This expanded structure reflects the complex and evolving nature of uncertainty in chronic, high-dependency treatments like hemodialysis. Although the MUIS-A remains widely used in China, it may not fully capture treatment-specific or sociocultural nuances relevant to long-term care. The UC-D&TS addresses this gap by providing greater conceptual granularity and cultural contextualization.

### 4.2 Theoretical and clinical implications

The five-factor structure of the Chinese UC-D&TS reflects the complex and multidimensional nature of illness uncertainty in hemodialysis patients. The emergence of “Alternative Treatment Uncertainty” and “Existential and Social Role Uncertainty” as distinct domains extends beyond traditional models and highlights the unique psychosocial burden faced by patients undergoing life-sustaining treatment. These dimensions are particularly relevant in the Chinese context, where family obligations, moral duty, and intergenerational expectations shape patients’ feelings of illness and treatment.

Our use of EGA not only corroborated the factor structure identified via EFA but also offered deeper insights into the interrelationships among items and dimensions. Centrality analysis identified four items—addressing uncertainty regarding future treatment, crisis preparedness, kidney transplant alternatives, and the life-extending value of HD—as the most influential nodes in the network. These items represent core cognitive-emotional challenges faced by HD patients and may serve as key targets for psychological intervention. From a clinical standpoint, interventions addressing these central uncertainties—through shared decision-making tools, counseling, or targeted health education—may yield disproportionately high returns in mitigating distress and improving patient adaptation.

A floor effect was observed for items 5, 7, 8, and 9. We posit that this is likely because the content of these items (e.g., understanding of the treatment regimen) represents well-established routines for patients on long-term regular dialysis, leading to a concentration of scores at the lower end. This indicates limited discriminatory power of these items for patients with very low uncertainty in these specific domains, and their interpretation warrants caution. Crucially, the absence of floor or ceiling effects for the total scale suggests that the instrument as a whole remains valid for assessing illness uncertainty in this patient population.

Moreover, the high test–retest reliability observed in this study suggests that the Chinese UC- D&TS is a stable and reliable tool for longitudinal monitoring. Given the increasing interest in digital health platforms and remote patient management, this tool may serve as a valuable psychometric component for risk stratification and personalized care algorithms in CKD management.

Beyond item-level refinements, the factor structure identified in the Chinese version diverged meaningfully from the original scale. This structural divergence reflects important cultural and contextual differences in how uncertainty is experienced and organized. In the original version of the UC- D&TS, five dimensions were proposed: Self-Uncertainty (Items 1–4), Uncertain Situations (Items 5–8), Uncertain Future (Items 9–11), Uncertainty of Treatment Outcomes (Items 12–14), and Information Uncertainty (Items 15–17). These dimensions were largely defined by temporal orientation and the locus of uncertainty (e.g., present vs. future, self vs. external circumstances).

In comparison, the five factors emerging from the Chinese version—Coping and Decision-Making Uncertainty, Outcome Expectancy Uncertainty, Treatment-Related Knowledge Uncertainty, Alternative Treatment Uncertainty, and Existential and Social Role Uncertainty—suggest a reorganization of these experiences into domains that more closely reflect the cultural and psychosocial realities of Chinese patients. Specifically, uncertainty is framed less around time and more around decision-making capacity, treatment consequences, knowledge sufficiency, and the existential implications of chronic illness for social identity and life purpose.

This structural shift suggests that Chinese patients may conceptualize uncertainty not primarily through temporally defined categories, but through culturally embedded constructs such as familial obligations, role expectations, and the perceived moral duty to persist with treatment. For instance, items related to daily functioning, emotional exhaustion, or treatment continuation—which were grouped under Self-Uncertainty or Uncertain Situations in the original model—were either excluded or redistributed into dimensions related to coping, values, and role-based concerns in the Chinese context.

Such differences highlight the necessity of cultural adaptation beyond literal translation. Conceptual and structural adjustments are essential to ensure that psychometric tools accurately capture the ways in which constructs like uncertainty are understood, expressed, and managed within specific cultural frameworks. This underscores the need for culturally sensitive measurement models in cross-cultural health research.

### 4.3 Strengths and innovations

This study offers several methodological and theoretical contributions. First, it fills a critical gap in psychometric tools tailored for HD patients in China, providing a culturally adapted and empirically validated instrument with strong structural and longitudinal properties. Second, the integration of modern network analysis techniques—rarely used in scale validation studies in nephrology—adds value by revealing non-linear item interdependencies and identifying intervention-prioritization targets. Third, the study applied a rigorous translation-back translation process, ensuring linguistic equivalence while also engaging patient feedback to enhance cultural appropriateness and acceptability.

### 4.4 Limitations and future directions

This study has several limitations. First, convenience sampling from tertiary hospitals in eastern and northeastern China may introduce selection bias, future studies should sample more broadly across different regions of China to enhance the generalizability and applicability of the findings. Second, while the sample size met psychometric criteria, future studies with larger, more diverse samples—including peritoneal dialysis and pre-dialysis CKD patients—are needed to enhance external validity. Third, the cross-sectional design limits conclusions about the scale’s responsiveness; longitudinal studies should assess sensitivity to clinical changes and predictive validity for psychological outcomes and treatment adherence. Lastly, a potential limitation of this study is the use of a self-report questionnaire, which may be prone to response bias due to factors such as social desirability or memory recall, future studies may consider incorporating additional data collection methods, such as clinician assessments or objective health indicators, to complement the self-reported data and mitigate potential biases.

Looking forward, future studies could explore the relationship between treatment uncertainty and other psychological factors, such as decisional regret (Xu et al., 2020). By examining how uncertainty influences decision-making processes and contributes to negative emotional outcomes like regret, researchers could gain deeper insights into how patients experience and cope with uncertainty in their treatment choices. Understanding these connections could further inform clinical strategies, improving both decision-making support and psychological interventions for hemodialysis patients.

Given the current trend of integrating digital technology into clinical work, future research should also explore the integration of the UC-D&TS into routine clinical workflows and digital health platforms for real-time monitoring and early psychological intervention (Zheng et al., 2025). Recent studies have shown that digital health solutions can play a key role in symptom management for hemodialysis patients, offering timely interventions for both physical and psychological symptoms, thereby improving overall patient outcomes. By leveraging technologies such as mobile health apps and telemedicine, clinicians can provide tailored, on-demand support to manage the symptoms and psychological burden associated with dialysis.

## 5 Conclusion

This study developed and validated the Uncertainty about Disease and Treatment Scale, a culturally grounded instrument capturing the multidimensional nature of uncertainty among Chinese hemodialysis patients. The five-factor structure reflects distinct aspects of patients’ uncertainty experiences and demonstrates strong reliability and validity. Compared to general illness uncertainty measures such as the MUIS-A, the UC-D&TS provides greater conceptual specificity and contextual relevance, offering a more precise assessment of disease and treatment-related uncertainty in chronic care settings. Its application can support individualized psychological interventions and inform clinical strategies aimed at improving patient adaptation and engagement.

## Data Availability

The raw data supporting the conclusions of this article will be made available by the authors, without undue reservation.
