# Supplementary material for: Psychometric properties and network analysis of the Chinese version of the uncertainty about disease and treatment scale for patients undergoing hemodialysis
Source: Front Psychol. 2026 Jan 5;16:1688355. doi: 10.3389/fpsyg.2025.1688355 (PMC12813107; doi:10.3389/fpsyg.2025.1688355)
Supplement: Supplementary file 1 [file Data_Sheet_1.pdf]

Revisions of items			
Items	issues	Revised items	Basis of revision
5	The expression of “ I am unsure about continuing with it.” was vague, and the patients were confused about whether to continue to treat the disease (including drugs and other treatments) or to continue hemodialysis treatment.	At times, when I become exhausted from the conditions associated with hemodialysis, I am unsure about continuing hemodialysis treatment	Clarify the problem formulation to make it easier for patients to understand.
13	In the context of Chinese culture, the "education" of a team is generally expressed as "training".	I am not certain whether training the treatment team can reduce the complications of hemodialysis.	After adjustment, it is more in line with the original intention and more in line with the Chinese cultural background.
17	The respondents thought that "vascular access" was a relatively technical term and difficult to understand, which should be specified in the entry.	I am not very certain about the information I have regarding my arteriovenous fistula or central venous catheter.	After adjustment, it is consistent with the original intention and easier for patients to understand

条目修改情况			
条目	问题	修订后	原因
5	“是否要继续下去”表述较为模糊，患者疑惑继续治疗疾病(包括药物等治疗方式)还是继续血液透析治疗	有时,当我因血液透析带来的症状而筋疲力尽时,我不确定是否要继续接受血透治疗	明确问题表述，使患者更易理解
13	在中国文化背景下，对团队的“教育”一般表述为“培训”	我不确定对治疗团队进行相关知识培训是否能减少血液透析的并发症	调整后更符合原意，同时更符合中国文化背景。
17	受访者认为“血管通路”较为术语，不易理解，应在条目中具体举例说明	我对自己所掌握的动静脉内瘘或中心静脉导管相关信息不太确定	调整后符合原意，同时患者更易理解

Chinese version of the Uncertainty about Disease and Treatment Scale

中文版疾病与治疗不确定性量表

	条目	完全不同意	有点不同意	中立	有点同意	完全同意
应对与决策不确定性	我不确定自己能否有效地运用策略来应对治疗带来的副作用					
	我不确定自己是否有能力做好准备去应对与血液透析相关的危机情况					
	每当我的治疗出现问题时，我发现很难轻易做出决定					
预期结果不确定性	我不确定血液透析是否是延长我寿命的合适方法					
	我不确定对治疗团队进行相关知识培训是否能减少血液透析的并发症					
	我不确定坚持治疗是否能延长寿命					
治疗相关知识不确定性	我不确定在未来的治疗中什么是不应该做的					
	关于未来的治疗，我有很多尚未解答的问题					
	我对自己所掌握的动静脉内瘘或中心静脉导管相关信息不太确定					
替代治疗认知与决策不确定性	我不确定我是否能接受其他治疗方法来治疗我的疾病					
	我不确定自己是否对肾移植有足够的了解					
	我不确定肾移植是否比血液透析好					
生活意义与社会角色不确定性	基于我目前正在接受的治疗方式，我不确定自己能否去追求人生目标和抱负					
	由于我的疾病或血液透析，我不确定将来自己能否在社会或家庭中拥有良好的地位					
	我正处于一种生活中没有什么能让我笃定依赖的状态					

The Uncertainty about Disease and Treatment Scale

	Item	Strongly disagree	Disagree	Neutral	Agree	Strongly agree
Coping and Decision-making Uncertainty	I am not certain if I can effectively utilize strategies to deal with the side effects associated with my treatment.					
	I am not certain of my ability to be prepared to handle crisis situations related to hemodialysis.					
	Whenever an issue arisesing my treatment, I find it difficult to make a decision about it easily.					
Outcome Expectancy Uncertainty	I am not certain whether hemodialysis is a suitable approach for extending my lifespan.					
	I am not certain whether training the treatment team can reduce the complications of hemodialysis.					
	I am not certain whether adhering to treatment will result in a longer lifespan.					
Treatment-related Knowledge Uncertainty	I am not certain of what not to do in the future for my treatment.					
	I have many unanswered questions about the future of my treatment.					
	I am not very certain about the information I have regarding my arteriovenous fistula or central venous catheter.					
Alternative Treatment Uncertainty	I am not certain if I can accept other treatment methods for my disease.					
	I am not certain whether I have enough information about kidney transplantation or not.					
	I am not certain if a kidney transplant is					

	better than hemodialysis.					
Existential and Social Role Uncertainty	I am not certain that I can pursue my life goals and aspirations based on the type of treatment I am receiving.					
	I am not certain about having a favorable social or family status in the future due to my disease or hemodialysis.					
	I am in a state where nothing in my life can be relied upon with certainty.					
